# Glial Activation and Expression of the Serotonin Transporter in Chronic Fatigue Syndrome

**DOI:** 10.3389/fpsyt.2018.00589

**Published:** 2018-11-16

**Authors:** Mami Noda, Masataka Ifuku, Md. Shamim Hossain, Toshihiko Katafuchi

**Affiliations:** ^1^Laboratory of Pathophysiology, Graduate School of Pharmaceutical Sciences, Kyushu University, Fukuoka, Japan; ^2^Department of Neuroinflammation and Brain Fatigue Science, Graduate School of Medical Sciences, Kyushu University, Fukuoka, Japan

**Keywords:** chronic fatigue syndrome, poly I:C, TLR3, IL-1beta, serotonin transporter

## Abstract

Fatigue is commonly reported in a variety of illnesses and has major impact on quality of life. Chronic fatigue syndrome (CFS) is a debilitating syndrome of unknown etiology. The clinical symptoms include problems in neuroendocrine, autonomic, and immune systems. It is becoming clear that the brain is the central regulator of CFS. For example, neuroinflammation, especially induced by activation of microglia and astrocytes, may play a prominent role in the development of CFS, though little is known about molecular mechanisms. Many possible causes of CFS have been proposed. However, in this mini-review, we summarize evidence for a role for microglia and astrocytes in the onset and the maintenance of immunologically induced CFS. In a model using virus mimicking synthetic double-stranded RNA, infection causes sequential signaling such as increased blood brain barrier (BBB) permeability, microglia/macrophage activation through Toll-like receptor 3 (TLR3) signaling, secretion of IL-1β, upregulation of the serotonin transporter (5-HTT) in astrocytes, reducing extracellular serotonin (5-HT) levels and hence reduced activation of 5-HT_1A_ receptor subtype. Hopefully, drug discovery targeting these pathways may be effective for CFS therapy.

## Introduction

CFS, also often described as myalgic encephalomyelitis, is characterized not only by severe and prolonged fatigue drastically impairing life quality, and much more attention has been paid in the past year than ever (1–6). CFS is observed even in children and adolescents (7). CFS is induced by impairment of neuronal–endocrine–immune interactions with various symptoms including circadian rhythm abnormalities (8, 9). Though systematic reviews were carried out recently, remarkable ways to reduce fatigue severity are still missing (1, 10–17). In the central nervous system (CNS), activation or dysfunction of glial cells are involved in various neurodegenerative disorders and psychiatric diseases or symptoms. It has been reported that neuroinflammation is present in widespread brain areas in CFS patients in a positron emission tomography (PET) study utilizing ^11^C-(*R*)-PK11195, a ligand for a translocator protein expressed in activated microglia or astrocytes (18). This finding suggests that activation of glial cells play an important role also in CFS. Though the brain regions involved in CFS still remain mysterious, a clinical study with CFS patients showed involvement of the prefrontal cortex (PFC) and frontal networks (19, 20). In addition, single-photon emission-captured tomography (SPECT) studies in CFS patients revealed that production of glutamate in the PFC was low (21). These findings suggest that fatigue sensation and dysfunction of the PFC are correlated, although the molecular/cellular mechanism is only partially known. Here, based on the recent reviews mentioned above, possible causes of CFS proposed during the last decade are summarized. Then, infection-related/immunological CFS is focused and how glial cells are involved in the development of fatigue sensation is described, giving a new insight as an alternative strategy for the treatment of CFS.

## Causes of CFS

CFS may be explained by both central and peripheral pathophysiological mechanisms (22), together with anatomical changes such as a decrease in gray matter volume in the brain (23). The etiology of CFS is still little advanced over the last decade (24). There are many potential causes, consisting of several categories. One is metabolic abnormalities or changes in neural activity, which were suggested by the studies with magnetic resonance spectroscopy (MRS) or magnetoencephalography (MEG) of muscles and brain in some CFS patients (25–28). The cellular metabolic abnormalities contain, for example, a decrease in nuclear factor erythroid-2-related factor 2 (Nrf2), antioxidant enzymes (superoxide dismutase 1&2, glutathione peroxidase, heme oxygenase-1, and catalase), and glycogen, and adenosine triphosphate (ATP). Though, it has been claimed that metabolic biomarkers for CFS were still not available (29), oxidative stress index was proposed as a potential biomarker for fatigue (30) and a diagnostic biomarker was proposed by comprehensive metabolomic analyses of blood samples obtained from patients (31, 32), for example, decreased plasma levels of carnitine-choline and phosphatidylcholine, while increased plasma levels of ceramide, triglyceride, and phosphatidylethanolamine. Dysfunction of the tricarboxylic acid (TCA) and urea cycles in CFS (33) and increase in lactic acid and blood urea nitrogen could also be the cause of CFS (34), which could become metabolite markers of CFS. Consequently, reduced cellular-energy availability and non-adaptive energy expenditure in CFS has been reported (35). Nevertheless, the reader should be aware that we do not know if these metabolic changes are a cause or a consequence of CFS. Recently, the question has been raised whether CFS is a metabolic disease or the result of disturbed homeostasis, and a multiplicity of physiological/pathophysiological factors related to CFS have been listed (36). On the other hand, a new concept called circadian metabolomics, the relationship between circadian rhythms and energy metabolism, has been proposed to explain circadian rhythm abnormalities in CFS patients (8).

The second possible mechanism is functional and structural abnormalities. For example, lymphatic dysfunction was postulated because cerebrospinal fluid drainage was beneficial in some CFS patients (37, 38). Other functional changes such as immune dysregulation, mitochondrial dysfunction (39), AMP-activated protein kinase, skeletal muscle cell acidosis are summarized in a recent review (29). In addition, recent genome-wide epigenetic analyses revealed different patterns of DNA methylation, which are related to cell signaling in immune system and could become possible diagnostic markers (40). Furthermore, changes in the amount and the size of circulating extracellular vesicles were also reported as potential biomarkers in CFS (41).

Finally, classical but still relevant studies revealed that CFS can occur after infection with viral and non-viral pathogens (42, 43), though virus infection as a cause of CFS has been questioned (44). Several lines of evidence indicate that pathogens entering the body induces expression of cytokines in the CNS (45, 46). Alternatively, CFS might be an autoimmune disease. Indeed, autoantibodies against various antigens, such as nuclear and membrane structures and neurotransmitters, have been identified in some CFS patients (47). The possible causes mentioned above are summarized in Table 1.

**Table 1:** Possible causes of CFS.

**METABOLIC ABNORMALITIES**			
Glycogen ATP Glutamate Carnitine-choline Phosphatidylcholine	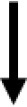	Lactic acid Blood urea nitrogen Ceramide Triglyceride Phosphatidylethanolamin	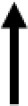
Irregularities in energy, amino acid, and nucleotide			
Disturbances in neurotransmitter-related pathways			
Abnormality in lipid metabolism, mitochondrial metabolites, and nitrogen metabolism			
Abnormal levels of cholesterol and vitamins			
**OXIDATIVE STRESS**			
Nrf2 SOD1,SOD2 GSH-Px H0-1 CAT	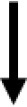		
**FUNCTIONAL AND STRUCTURAL ABNORMALITIES**			
Glymphatic dysfunction			
Decrease of gray matter volume			
Immune dysregulation			
Mitochondrial dysfunction			
TCA and urea cycle dysfunction			
Changes in AMP-activated protein kinase			
Skeletal muscle cell acidosis			
Different DNA methylation patterns			
Increased amount and reduced size of circulating extracellular vesicles			
**INFECTION**			
Viral and non-viral pathogens			
**AUTOIMMUNE DISEASE**			
Autoantibodies against various antigens			

*Nrf2, nuclear factor erythroid-2-related factor 2; SOD, superoxide dismutase; GSH-Px, glutathione peroxidase; HO-1, heme oxygenase-1; CAT, catalase; TCA, tricarboxylic acid*.

In addition to immune dysfunction, activation of the immune system occurs in both infection- or autoantigens-related CFS. In the CNS, glial cells, especially microglial cells, play an important role the immune system. Therefore, inflammation-induced functional changes in neuron-glial interaction in the CNS, affecting peripheral organs, could be fundamental cause of CFS.

## Poly-I:C (polyinosinic-polycytidylic acid sodium salt)-induced CFS animal model

In the case of infection-related CFS, it is well-known that inflammatory cytokines in the brain can affect the sympathetic nervous system, the hypothalamic–pituitary–adrenal (HPA) axis, peripheral cellular immunity, learning and memory, and behavioral activity (46, 48, 49). It is most likely that cytokines might play a role in the development of CFS initiated/triggered by viral/non-viral pathogens. To investigate this, systemic injection of poly-I:C, virus-mimicking synthetic double-stranded RNA, is a useful approach. Interestingly, poly-I:C injection causes a sustained decrease in spontaneous running wheel activity that is accompanied by enhanced expression of interferon-α (IFN-α), IL-1β, and 5-HTT in the rat brain (48, 50). It must be noted that in this experimental model the reduced activity is not attributable to sickness behavior or depression, as the activity decrease is still observed after the poly-I:C-induced acute-phase responses, such as fever and adrenal responses, were abolished (51). Curiously, however, open field tests showed no differences from controls (52).

## Disruption of blood-brain barrier (BBB) by poly-I:C

A question arises whether or not intraperitoneal (i.p.) injection of poly-I:C causes breakdown of BBB. There is evidence that poly-I:C increases BBB permeability in a rat model of CFS (48). The breakdown of the BBB can be assessed by quantification of extravasated Evans Blue in the brain (53–56). With this method, extravasated Evans Blue content in the brain indeed increases at 12 and 24 h after poly-I:C injection. Interestingly, the relative expression levels of microglial activation marker mRNAs began to increase 12 h after poly-I:C administration, which showed a similar time course in the change of BBB permeability (48). This may partly, if not entirely, explain how i.p. injection of poly I:C induces morphological activation of microglia in the brain.

## Involvement of toll-like receptor 3 (TLR3) and activation of astrocytes in poly-I:C signaling

TLR3 recognizes viral double-stranded (ds)RNA, mimicked by poly-I:C. As a byproduct of viral replication, dsRNA serves as the signature molecule for viral infection via TLR3 (57). Poly I:C, as a TLR3 agonist, induces suppression of spontaneous activity in rats (51). The involvement of TLR3 on West Nile virus infection, causing lethal encephalitis, has also been reported (58). Viral infections emerge as important events in the etiology of CNS damage, and it was reported that dsRNA/TLR3-activated astrocytes initiate a battery of rapid innate immune responses (57). However, activation of astrocytes turned out to be secondary to TLR3 signaling in the CNS (48).

## Activation of microglia/and macrophages with IL-1β expression in CFS model animal

IL-1β expression is deeply associated with poly-I:C-induced fatigue in rats (48, 51). IL-1β is the typical pro-inflammatory cytokines produced by activated microglia. It was indeed shown that direct application of poly-I:C to primary cultured rat microglia enhances IL-1β expression (48). Therefore, it is likely that i.p. injection of poly-I:C induces IL-1β expression in microglia/macrophages at least partly via direct action of poly-I:C. TLR agonists also upregulate other inflammatory cytokines such as IL-4, IL-5, IL-10, IL-13, and TNF-α (59). However, these cytokines are not often analyzed in CFS animal models. The importance of IL-1β in CFS was proved using a neutralizing antibody against IL-1β (48) or IL-1 receptor antagonist (51) injected into the lateral ventricle of rats.

Instead of blocking IL-1β, prevention of microglial activation may also be useful as well. Pretreatment with minocycline, an inhibitor of microglia/macrophage activation, prevented the increased expression of microglial IL-1β and induction of CFS (48). Since activation of microglia is the key event of other neurological disorders, many beneficial effects of minocycline have recently been reported. The inhibitory effects of minocycline on the neurological diseases, however, seem to be only effective by pretreatment. For example, once microglial cells in spinal cord were activated by sciatic nerve injury, minocycline could not inhibit the neuropathic pain (Akimoto et al., unpublished data). In addition, microglial activation at the onset and the chronic phase of neurological diseases seems quite different. For example, after traumatic brain injury, minocycline reduced chronic microglial activation, while a marker of neurodegeneration increased (60). These findings suggest that microglial activation may have a reparative effect in the chronic phase of neurological disorders, presumably including CFS. The function of activated microglia/macrophages may depend on the time and microenvironment of the brain pathology.

## Microglial IL-1β, but not IFN-α, upregulates expression of 5-HTT in astrocytes

Glial interaction between microglia and astrocytes is crucial for neuronal function in the brain (61, 62). The poly-I:C-induced upregulation of 5-HTT in astrocytes, not in microglia, in the brain of CFS is also the case. The direct effect of poly-I:C is actually on microglia, which then affect astrocytes, because the upregulated expression of 5-HTT in the brain of poly-I:C-injected rats was abolished by pretreatment of minocycline, an inhibitor of microglial activation (48). Therefore, activation of microglia is the trigger for upregulation of 5-HTT in astrocytes.

The messenger from microglia to astrocytes is IL-1β. Among pro-inflammatory cytokines or other factors released from activated microglia (61), IL-1β plays a pivotal role in CFS. The interaction between microglial IL-1β and astrocytic 5-HTT was proved by using IL-1 receptor antagonists. Upregulation of 5-HTT in astrocytes from poly-I:C-injected rat brain was completely blocked by IL-1 receptor antagonists. Furthermore, it was shown that IL-1β directly upregulates the expression of 5-HTT in primary cultured rat astrocytes (48). The function of 5-HTT in astrocytes has been already demonstrated in the rat brain (63).

On the other hand, treatment with IFN-α, one of the factors upregulated by TLR agonists (50), did not affect 5-HTT expression in primary cultured astrocytes (48). Though other factors have not yet been fully investigated, IL-1β is probably the most important factor among poly-I:C-related factors.

## Reduction of 5-HT and induction of fatigue

5-HTT expressed in astrocytes is sensitive to antidepressants and contributes to control extracellular 5-HT concentration, i.e., 5-HT taken up by astrocytes is inactivated through deamination by monoamine oxidase A, then scavenged into the cerebrospinal fluid. On the other hand, 5-HT taken up by neurons is re-utilized as a neurotransmitter (64, 65). The microdialysis experiment demonstrated that administration of poly-I:C decreased the extracellular levels of 5-HT, which was abolished by imipramine, a non-selective 5-HT reuptake inhibitor (50). Therefore, it is possible that functional 5-HTT overexpressed on astrocytes scavenges 5-HT, thereby leading to the reduction in the 5-HT levels following poly-I:C injection. Additionally, the importance of the 5-HT_1A_ receptor, but not 5-HT_2_, 5-HT_3_ or dopamine D3 receptor, has been suggested (50, 66). Considering these information, a specific agonist for the 5-HT_1A_ receptor, as well as 5-HTT reuptake inhibitor, would be promising therapeutic drugs for CFS.

## Conclusion

**Figure 1:**
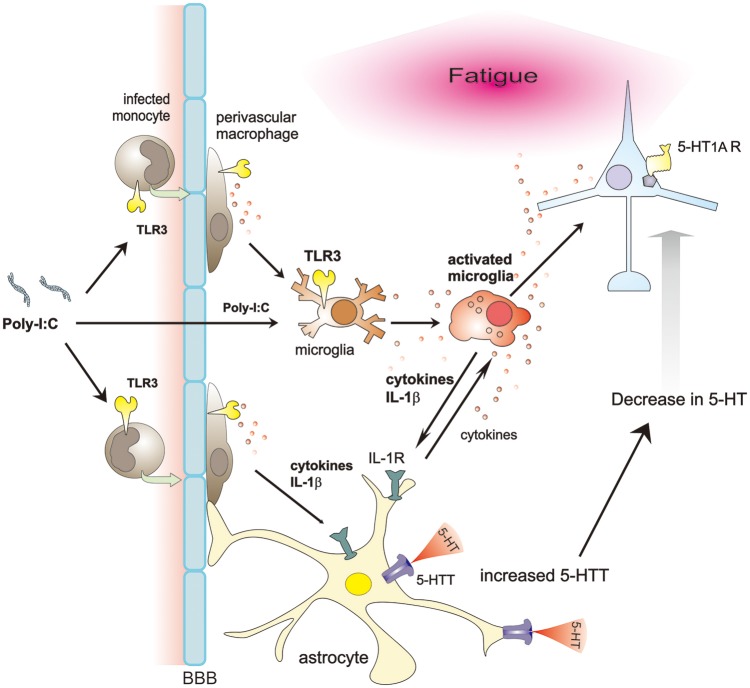
Proposed schema on glia-neuron interaction in a model of immunologically induced fatigue. Poly-I:C, a synthetic double-stranded RNAs, increases BBB permeability, entering the CNS and activating microglia/macrophages through TLR3 signaling. Activated microglia/macrophages secrete various cytokines. Among them, IL-1β upregulates expression of 5-HTT in astrocytes. Due to increased expression of 5-HTT, extracellular 5-HT decreases, impairing the 5-HT signaling, especially via 5-HT_1A_ receptors, thereby possibly inducing fatigue sensation.

Neuronal function is altered by glial cells in many ways. In a model of immunologically induced fatigue, poly-I:C increases BBB permeability and activates microglia/macrophages through TLR3 signaling. Activated microglia/macrophages secrete IL-1β, which induces 5-HTT expression in astrocytes, reducing extracellular 5-HT levels, and finally less activation of 5-HT_1A_ receptor. Consequently, these cascades possibly produce fatigue sensation (Figure 1). Therefore, inhibition of microglial IL-1β and astrocytic 5-HTT, or specific activation of 5-HT_1A_ receptor could be promising ways to ameliorate the various symptoms of CFS, at least in infection-related cases.

## Author contributions

MN wrote the manuscript. MI performed experiments and contributed writing manuscript. MSH helped experimenting and editing manuscript. TK used to manage the experiments and wrote the abstract draft.

### Conflict of interest statement

The authors declare that the research was conducted in the absence of any commercial or financial relationships that could be construed as a potential conflict of interest.
